# Multilocus variable-number tandem-repeat analysis of clinical isolates of *Aspergillus flavus* from Iran reveals the first cases of *Aspergillus minisclerotigenes* associated with human infection

**DOI:** 10.1186/1471-2334-14-358

**Published:** 2014-07-01

**Authors:** Parvin Dehghan, Tien Bui, Leona T Campbell, Yu-Wen Lai, Nai Tran-Dinh, Farideh Zaini, Dee A Carter

**Affiliations:** 1 Department of Parasitology & Mycology, Faculty of Medicine, Isfahan University of Medical Sciences, Isfahan, Iran; 2 School of Molecular Bioscience, The University of Sydney, Sydney, NSW, Australia; 3 CSIRO Animal, Food and Health Sciences, Riverside Corporate Park, North Ryde, Sydney, NSW 2113, Australia; 4 Department of Parasitology & Mycology, School of Public Health, Tehran University of Medical Sciences, Tehran, Iran

## Abstract

**Background:**

*Aspergillus flavus* is intensively studied for its role in infecting crop plants and contaminating produce with aflatoxin, but its role as a human pathogen is less well understood. In parts of the Middle East and India, *A. flavus* surpasses *A. fumigatus* as a cause of invasive aspergillosis and is a significant cause of cutaneous, sinus, nasal and nail infections.

**Methods:**

A collection of 45 clinical and 10 environmental *A. flavus* isolates from Iran were analysed using Variable-Number Tandem-Repeat (VNTR) markers with MICROSAT and goeBURST to determine their genetic diversity and their relatedness to clinical and environmental *A. flavus* isolates from Australia. Phylogeny was assessed using partial β-tubulin and calmodulin gene sequencing, and mating type was determined by PCR. Antifungal susceptibility testing was performed on selected isolates using a reference microbroth dilution method.

**Results:**

There was considerable diversity in the *A. flavus* collection, with no segregation on goeBURST networks according to source or geographic location. Three Iranian isolates, two from sinus infections and one from a paranasal infection grouped with *Aspergillus minisclerotigenes*, and all produced B and G aflatoxin. Phylogenic analysis using partial β-tubulin and calmodulin sequencing confirmed two of these as *A. minisclerotigenes,* while the third could not be differentiated from *A. flavus* and related species within *Aspergillus* section *flavi*. Based on epidemiological cut-off values, the *A. minisclerotigens* and *A. flavus* isolates tested were susceptible to commonly used antifungal drugs.

**Conclusions:**

This is the first report of human infection due to *A. minisclerotigenes,* and it raises the possiblity that other species within *Aspergillus* section *flavi* may also cause clinical disease*.* Clinical isolates of *A. flavus* from Iran are not distinct from Australian isolates, indicating local environmental, climatic or host features, rather than fungal features, govern the high incidence of *A. flavus* infection in this region. The results of this study have important implications for biological control strategies that aim to reduce aflatoxin by the introduction of non-toxigenic strains, as potentially any strain of *A. flavus,* and closely related species like *A. minisclerotigenes*, might be capable of human infection.

## Background

*Aspergillus flavus,* notorious for its production of the toxic and carcinogenic mycotoxin aflatoxin, is also capable of affecting human health through infection and pathogenic growth. While life-threatening disseminated disease only occurs in people with relatively severe immunocompromise, *A. flavus* can cause superficial infections of the skin and nails, and localized infection within the sinuses in otherwise healthy people. These infections are particularly prevalent in warmer regions of the world, particularly the Middle East, where *A. flavus* is the dominant pathogen in fungal sinusitis and keratitis [1].

Although encountered worldwide, the growth of *A. flavus* is enriched by certain agricultural crops including corn, peanuts, pistachio and cotton [2]. *A. flavus* is a rapidly growing fungus, producing large numbers of microscopic asexual conidia that are readily dispersed and are the infectious propagule. Population genetic analyses suggest that *A. flavus* has a largely panmictic population structure [3,4], however there is also evidence of clonally derived outbreaks associated with human infection [5].

Iran has a largely agricultural economy and a warm climate that favors the growth of thermotolerant fungi like *A. flavus*, which has been isolated from certain crops and associated soils [6]. *A. flavus* is the leading cause of chronic fungal rhinosinusitis in Iran [7], and it has been isolated from the air, internal surfaces and surrounding soils of a number of Iranian hospitals [1]. Population genetic studies of the hospital-related isolates using RAPD analysis found these divided into two distinct groups, however no standard strains were included and it was not possible to compare these with other global isolates. Denghan et al. [8] reported the cultural characteristics and mycotoxin profiles of a set of 55 clinical and environmental isolates from Iran belonging to the *A. flavus* species complex. All but one of the clinical isolates was *A. flavus*, relatively few (11.1%) were aflatoxigenic, and one clinical isolate was *A. oryzae*[8].

The aim of the current study was to examine the genetic diversity and population structure of the clinical *A. flavus* isolates collected by Denghan et al. [8]. Multilocus variable-number tandem repeat analysis (MLVA) was used to compare these with type strains of *A. flavus, A. parasiticus*, *A, sojae* and *A. oryzae,* and with a selection of clinical and environmental isolates from Australia*,* including one isolate of *A. minisclerotigenes*. We show here that clinical Iranian isolates are diverse and related to their environmental counterparts and to Australian isolates, with one isolate indistinguishable from an isolate from Australia. We report the first isolation of A. *minisclerotigenes* from Iran, and the first association of this species with clinical disease, with isolates obtained from one paranasal and one sinus infection.

## Material and methods

### Isolates

Seventy isolates were included in an initial analysis of the diversity of Iranian isolates. These included 45 clinical *A. flavus* isolates, one clinical *A. oryzae* isolate and 10 non–clinical *A. flavus* isolates from Iran, along with 4 clinical and 2 non-clinical *A. flavus* isolates and one non-clinical *A. minisclerotigenes* isolate from Australia. Clinical isolates from Iranian patients were obtained during standard patient care at participating clinics, where specimens and biopsies of the lesions were referred by physicians to the Mycology Reference Laboratory in School of Public Health, Tehran University of Medical Sciences for diagnosis of the etiologic agents of infection. For identification of fungal elements in tissues, specimens were treated by potassium hydroxide (KOH) and observed by direct microscopy. Portions of each specimen were inoculated on Sabouraud dextrose agar and cultures were incubated at 25°C. Positive KOH preparations showed dichotomous septate branching hyphae in tissue lesions. Identification of isolates as *A. flavus* was confirmed by colony morphology on Czapeck agar media and by microscopy following culture on malt extract agar (MEA), as reported previously [8]. As isolations and diagnoses were performed during routine clincial procedures there was no requirement for ethical approval. Type strains of *A. flavus, A. parasiticus, A, sojae* and *A. oryzae* were also included in the isolate collection (Table 1).

**Table 1:** Isolates included in study with VNTR alleles

**Strain designation**	**Morphological (molecular**^**1**^**) identification**	**Country of origin**	**Source**	**Aflatoxin production**	**VNTR allele size (bp)**					**MLV genotype**	**MAT allele**
					**AFPM1**	**AFPM4**	**AFPM5**	**AFPM6**	**AFPM7**		
***Iranian clinical and non-clinical isolates***											
65822	*A. flavus*	Iran	Sinus	Neg	122	205	215	345	256	**1**	
65712	*A. flavus*	Iran	Sinus	Neg	122	226	213	345	256	**2**	
9573	*A. flavus*	Iran	Sputum	Neg	122	219	215	345	193	**3**	
2117	*A. flavus*	Iran	Nail	Neg	122	217	215	345	250	**5**	
1608	*A. flavus*	Iran	Nail	Neg	122	195	215	345	248	**7**	
65895	*A. flavus*	Iran	Neck	Neg	122	220	215	345	193	**8**	
2037	*A. flavus*	Iran	Nail	Neg	122	213	213	345	239	**9**	
65770	*A. flavus*	Iran	Sinus	Neg	122	215	215	435	245	**10**	
1097	*A. flavus*	Iran	Nail	Neg	null	220	215	345	248	**11**	
65836	*A. flavus*	Iran	Sinus	Neg	122	207	215	345	258	**12**	
65811	*A. flavus*	Iran	Sinus	Neg	122	220	215	345	300	**13**	
65483	*A. flavus*	Iran	Sinus	Neg	122	213	215	345	248	**14**	
2141	*A. flavus*	Iran	Nail	Neg	122	220	213	345	254	**15**	
65829	*A. flavus*	Iran	Lung	Neg	122	205	215	345	256	**1**	
66041	*A. flavus (A. minisclerotigenes)*	Iran	Paranasal	Pos (B + trace G)	119	201	237	342	219	**16**	2
2165	*A. flavus*	Iran	Nail	Neg	122	211	213	345	256	**17**	
65938	*A. flavus*	Iran	Sinus	Pos	122	207	215	345	254	**18**	
65931	*A. flavus*	Iran	Sinus	Neg	122	201	213	345	256	**19**	
1698	*A. flavus*	Iran	Nail	Neg	122	209	213	345	245	**20**	
66246	*A. flavus*	Iran	Paranasal	Neg	122	228	215	347	263	**21**	
66204	*A. oryzae*	Iran	Sinus	Neg	122	203	213	345	256	**22**	
65760	*A. flavus*	Iran	Sinus	Neg	122	219	213	null	254	**23**	
65838	*A. flavus*	Iran	Lung (ball)	Neg	122	197	213	345	241	**24**	
292	*A. flavus*	Iran	Nail	Neg	122	211	215	345	256	**25**	
2666	*A. flavus*	Iran	Nail	Neg	122	215	215	345	254	**26**	
65817	*A. flavus*	Iran	Sinus	Pos	122	215	215	345	256	**27**	
65728	*A. flavus (?)*	Iran	Sinus	Pos (B + trace G)	123	197	211	348	null	**28**	1
4521	*A. flavus*	Iran	Nail	Neg	122	215	215	345	248	**29**	
2328	*A. flavus*	Iran	Nail	Neg	122	222	215	347	252	**30**	
65805	*A. flavus*	Iran	Sinus	Neg	122	205	215	345	256	**1**	
489	*A. flavus*	Iran	Nail	Neg	122	215	215	345	248	**29**	
66262	*A. flavus*	Iran	Sinus	Neg	122	211	213	345	193	**31**	
66086	*A. flavus*	Iran	Paranasal	Neg	122	203	213	349	246	**32**	
66161	*A. flavus (A. minisclerotigenes)*	Iran	Sinus	Pos (B + G)	119	201	235	346	219	**33**	1
66150	*A. flavus*	Iran	Paranasal	Neg	122	205	213	345	193	**34**	
66666	*A. flavus*	Iran	Endocard	Neg	122	195	213	345	256	**35**	
1750	*A. flavus*	Iran	Nail	Neg	122	199	213	345	253	**37**	
66137	*A. flavus*	Iran	Paranasal	Neg	122	209	213	345	245	**20**	
65718	*A. flavus*	Iran	Sinus	Neg	122	225	213	346	256	**38**	
64726	*A. flavus*	Iran	Sinus	Neg	122	215	215	345	248	**29**	
64729	*A. flavus*	Iran	Sputum	Neg	122	195	215	345	248	**7**	
65932	*A. flavus*	Iran	Sinus	Neg	122	195	215	345	252	**39**	
65717(1)	*A. flavus*	Iran	Sinus	Neg	122	213	213	345	193	**40**	
65717(2)^2^	*A. flavus*	Iran	Sinus	Neg	122	213	213	345	193	**40**	
ES1	*A. flavus*	Iran	Pistachio	Neg	122	215	215	345	248	**29**	
ES2	*A. flavus*	Iran	Pistachio	Neg	122	215	215	345	264	**41**	
65841	*A. flavus*	Iran	Paranasal	Neg	122	218	215	345	256	**44**	
65848	*A. flavus*	Iran	Sputum	Neg	122	217	213	345	241	**45**	
TR	*A. flavus*	Iran	Soil	Pos	122	205	215	347	null	**42**	
NTR	*A. flavus*	Iran	Soil	Neg	122	219	215	345	null	**43**	
65806	*A. flavus*	Iran	Environment	Neg	122	219	215	345	193	**3**	
65824	*A. flavus*	Iran	Environment	Neg	122	205	215	345	256	**1**	
65773	*A. flavus*	Iran	Environment	Neg	122	219	213	347	249	**46**	
66130	*A. flavus*	Iran	Environment	Neg	122	205	213	345	193	**34**	
65844	*A. flavus*	Iran	Environment	Neg	122	228	213	345	256	**47**	
4941	*A. flavus*	Iran	Environment	Neg	122	195	215	345	193	**48**	
***Australian clinical and non-clinical isolates***											
A5	*A. flavus*	Australia	Paranasal	Neg	122	199	213	null	239	**49**	
C3	*A. flavus*	Australia	Ear	Neg	122	197	213	345	241	**24**	
E3	*A. flavus*	Australia	Sputum	Neg	122	188	213	345	252	**53**	
E4	*A. flavus*	Australia	Sputum	Neg	122	207	215	345	260	**54**	
FRR 5305^3^	*A. flavus*	Australia	Soil	Pos	122	196	215	344	243	**50**	
FRR 5309	*A. minisclerotigenes*	Australia	Soil	Neg	119	186	271	342	219	**51**	1
FRR 5314	*A. flavus*	Australia	Soil	Neg	122	188	213	344	249	**52**	
***Type strains***^**3**^											
NCPF 2008	*A. flavus*	Unknown	Ear	Neg	122	203	213	345	239	**4**	1 + 2
PTCC 5006	*A. flavus (A. tamarii)*	Iran		Neg	122	159	213	343	221	**61**	1
JCM 2061	*A. flavus*	Japan		Neg	122	215	213	345	244	**6**	1 + 2
IMI 126842	*A. oryzae*	USA	Chinese soy sauce	Neg	122	189	215	345	275	**65**	2
ATCC 15517	*A. parasiticus*	Japan		Pos	116	181	244	350	241	**63**	
NRRL 255	*A. parasiticus*	USA	Soil	Pos	116	181	245	350	235	**62**	1
IMI 191303	*A. sojae*	Japan		Neg	116	181	230	363	231	**64**	
***A. flavus "Group II" isolates***^***4***^											
FRR 2755	*A. flavus (A. minisclerotigenes)*	Australia	Peanuts	Neg	119	196	287	342	219	**55**	
FRR 4472	*A. flavus (A. minisclerotigenes)*	Australia	Environment	Pos (B + G)	119	186	341	342	219	**60**	1
FRR 4086	*A. flavus (A. minisclerotigenes)*	Australia	Environment	Neg	119	186	271	342	219	**66**	1
LA2-5 SB	*A. flavus (?)*	USA	Soil	Pos (B)	119	184	215	348	235	**56**	1
MS22-41 SB	*A. flavus (?)*	USA	Soil?	Pos (B)	119	186	217	348	271	**57**	
FRR 3384	*A. flavus (A. minisclerotigenes)*	Africa	Sunflower seeds		119	188	319	344	219	**58**	2
FRR 3382	*A. flavus (A. minisclerotigenes)*	Africa	Sunflower seeds		119	186	282	342	217	**59**	
M40 No1	*A. flavus (A. minisclerotigenes)*	South America	Environment	Neg	119	186	303	342	219	**67**	1
MEA 511	*A. flavus (A. minisclerotigenes)*	South America	Environment	Pos (B + G)	119	186	319	342	219	**68**	1
MEA 352	*A. flavus (A. minisclerotigenes)*	South America	Environment	Pos (B + G)	119	186	267	342	219	**69**	1

^1^Molecular identification based on MLVA and on b-tubulin and calmodulin phylogeny; only listed if different to morphological identification.

^2^From same infection as 657 17(1).

^3^FRR: Food Research Laboratory, CSIRO (Australia); PTCC: Persian Type Culture Collection (Iran); JCM: Japanese Collection of Microorganisms; NCPF: National Collection of Pathogenic Fungi (UK) IMI: International Mycological Institute (UK) ATCC: American Type Culture Collection (USA); NRRL: National Regional Reference Laboratory (USA).

^4^VNTR data taken from [3] with 3 bp subtracted from each locus size to account for modified primers.

As some of the Iranian isolates appeared similar to an isolate that had previously been characterised as belonging to *A. flavus* Group II (now formally described as *A. minisclerotigenes*) [9,10] ten additional Group II-like isolates from Australia, the USA and Africa were added to the analysis [3]. Two additional type strains of *A. oryzae* were also included, bringing the total number of isolates to 80. All isolates along with their species name and origin are listed in Table 1.

Morphological identification of the Iranian isolates was performed using standard methods of direct examination and culture, as described in Dehghan et al. [8]. Photomicrographs were produced using a Zeiss Axioscop microscope fitted with Nomarski Interference Contrast optics, a Zeiss Axiocam and Zeiss AxioVision software (Carl Zeiss, Sydney).

### Detection of aflatoxin production

Toxin production by isolates was assessed by inoculating on coconut cream agar (50% coconut cream and 1.5% agar) for 3 days at 30°C and observing colonies under long wavelength (365 nm) ultraviolet light. Intense fluorescence around the fungal colonies was presumptive evidence that a strain could produce aflatoxin. Blue/violet fluorescence indicated the production of B aflatoxin only, while a blue/white fluorescence indicated production of both B and G aflatoxins [11].

Toxin production by strains with *Aspergillus minisclerotigenes*-like MLV profiles was further assessed using LC-MS. Following growth of cultures overnight in coconut cream broth (50% coconut cream in deionised water), 10 mL of the culture supernatant was removed and centrifuged at 10,000 rpm for 10 min. A 5 mL aliquot of the resulting supernatant was subjected to solid phase extraction using a C-18 cartridge (900 mg, 4 mL reservoir, Alltech, Australia) under vacuum. Elution was achieved using 5 mL 100% acetonitrile. The eluants were reduced to 200–300 μL and were then re-constituted to 1 mL with 20% acetonitrile. LC-MS/MS analysis was conducted on a Quantum triple stage quadrapole (TSQ) mass spectrometer (Thermo Fisher Scientific, Waltham, MA, USA), equipped with a quaternary solvent delivery system, a column oven, a photo-diode array detector and an auto-sampler. A 20 μL aliquot of each sample was injected and separated on a Hydro Synergi C18 analytical column (150 mm × 2.0 mm, 5 μm particle size, Phenomenex, NSW, Australia) at 30ºC. The following solvents with a flow rate of 200 μL/min were used: A, 0.2% formic acid in purified water; and B, 0.2% formic acid in acetonitrile. The elution profile was a linear gradient for solvent B of 20% to 100% in solvent A over 18 minutes. Ions were generated using an electrospray source in the positive mode under conditions optimised for aflatoxin B1. Ions were acquired in the selected reaction monitoring mode for each aflatoxin as: B1 » 313 (241,285); B2 » 315 (259,287); G1 » 329 (200,243); G2 » 331 (313, 245). Quantification was achieved using external calibration curves.

### Extraction of DNA

DNA extraction was performed on samples grown on CYA medium for 5 days at 30°C, and was based on the method of [12]. Approximately 0.5 g of mycelia was scraped from the surface of the fungal colony and collected into a 1.5 ml microcentrifuge tube. Tubes containing mycelia were immediately frozen with liquid nitrogen, and the mycelia were ground to a fine powder with a small plastic pestle (VWR Scientific) and then re-suspended in 400 μl lysis buffer (50 mм Tris–HCl, 100 mм NaCl, 5 mм EDTA, 1% SDS). Five microlitres of proteinase K (20 mg ml^-1^) was added, and the mixture was incubated at 60°C for 30 min and then incubated at 40°C for 2 h. Following incubation, 112 μl of 5 M NaCl and 1/10 vol. CTAB (Cetyl Tri-methyl Amonium Bromide) were added to the tube with incubation at 65°C for 10 min. An equal volume of chloroform isoamyl alcohol (24:1) was then added and tubes were left on ice for 30 min. The mixture was centrifuged at 12,000 *g* for 10 min and the supernatant was transferred to another tube, mixed with 200 μl of 3 M sodium acetate (pH 5.2) and incubated overnight on ice. The following day, tubes were centrifuged at 2,000 *g* for 10 min at 4°C, the supernatant was transferred to a new tube, 0.55 vol. isopropanol was added and DNA was precipitated by centrifugation at 12,000 *g* at 4°C for 5 min. The DNA pellet was washed by cold ethanol, dried and resuspended in 50 μl TE buffer containing 10 μg/ml RNase. DNA was electrophoresed in a 0.7% agarose gel and visualized by ethidium bromide staining and UV transillumination. Concentrations were standardized to approximately 100 μg/mL by visual comparison with known standards.

### PCR amplification

VNTR markers AFPM1-7 were used to analyze the genetic relatedness of isolates [13]. Difficulties were encountered in amplifying some of the strains using primers AFPM2 and AFPM3, therefore only primers AFPM1 and AFPM4-7 were used (Table 2). Amplifications were carried out as described in [13].

**Table 2:** Characteristics of VNTR markers in Iranian/Australian population

**Locus**	**Repeat sequence**	**No. of alleles/genotypes**	**Simpson’s index of diversity**
AFPM-1	(CCA)_n_(CTA)_n_(CCA)_n_	3	0.1205
AFPM-4	(CA)_n_	21	0.9406
AFPM-5	(AG)_n_AC(AG)_n_	5	0.5506
AFPM-6	(GT)_n_	8	0.3682
AFPM-7	(AC)_n_	20	0.9064
*All loci*		*53*	*0.9913*

### Phylogenetic and mating type analysis

Phylogenetic analysis of Iranian isolates grouping with *A. minisclerotigenes* was done using partial β-tubulin and calmodulin gene sequences. Sequences were amplified from selected isolates using MyTaq Red Master Mix (Bioline, UK) according to manufacturer’s instructions and previously published primers for β-tubulin (bt2a and bt2b, [14]) and calmodulin (cf1 and cf4 [15]). Sequences were edited in Geneious Pro 6.1.6 (created by Biomatters; available at http://www.geneious.com/) and were aligned with published *Aspergillus* section *flavi* sequences [10]. Phylogenetic analysis was undertaken using maximum likelihood with the PAUP 4.0 plug-in for Geneious. 1000 replicates were used to determine bootstrap support values. Accession numbers are provided in on the relevant phylogenetic trees. All taxa information, matrices and trees have been made available through TreeBASE at the following URL: http://purl.org/phylo/treebase/phylows/study/TB2:S15946.

Mating type analysis was performed on all Group II/*A. minisclerotigenes*-like isolates in our collection along with selected type strains. Mating type was determined by amplification using MAT1-1 and MAT1-2 specific primers (M1F and M1R, M2F and M2R; [16]) in separate reactions. PCR amplification was performed using MyTaq Red Master Mix (Bioline, UK) according to manufacturer’s instructions.

### Antifungal susceptibility testing

The susceptibility of *A. minisclerotigenes* isolates in our collection was assessed for four antifungal agents that are used to treat *A. flavus* infections: Itrconazole (ITZ), voriconazole (VRZ), amphotericin B (AMB) and caspofungin (CAS). Minimum inhibitory concentrations (MICs) were determined using a modified version of the CLSI microdilution protocol for filamentous fungi (M38-A2) and yeasts (M27-A2) [17,18]. Drugs were diluted in 100% DMSO (ITR and VOR) or sterile water (AMB and CAS) to stock concentrations of 1,600 μg/mL. Two-fold serial dilutions were made in RPMI-1640 medium across flat-bottom 96 well microtitre plates to give drug concentrations ranging from 0.015 – 8 μg/mL. Drug free and cell free controls were included.

MIC reference strains *A. flavus* ATCC 204304 and *Aspergillus fumigatus* ATCC 204305 were purchased from the American Type Culture Collection. All *Aspergillus* strains were grown on sabouraud dextrose agar (SDA) and were incubated at 37°C for 5 – 7 days to obtain good sporulation. Spores were collected by flooding agar plates with 5 mL of sterile water containing 0.1% tween 20 and were counted with a haemocytometer and adjusted to 2 – 5 × 10^5^ spores/mL. Suspensions were diluted 1:50, and 100 μL was added to 100 μL of diluted drug in each well of the microtitre plate, giving a final spore concentration of 2 – 5 × 10^3^ spores/mL. *Candida krusei* ATCC 6258 was included as an additional MIC control. Following growth on SDA at 30°C for 48 hr, single colonies of *C. krusei* were suspended in sterile water and adjusted to a concentration of 1 – 5 × 10^6^ cells/mL. Microtitre plates were prepared as above and inoculated with 100 μL of a 1:1,000 dilution of the working suspension, giving a final cell concentration of 0.5 – 2.5 × 10^3^ cells/mL.

All plates were incubated for 48 hr at 37°C. Following the CLSI protocol, *Aspergillus* MICs for ITR, VOR and AMB were read as the drug concentration causing 100% growth inhibition compared to the drug free control. For CAS, the minimum effective concentration (MEC) was the first drug concentration to inhibit normal hyphal development. For *C. krusei*, inhibition was read at 100% for AMB and at 80% for the remaining drugs. All MIC testing was performed in triplicate on separate days.

### Data analysis

#### Analysis of genetic diversity

Simpson’s Index of Diversity was used to evaluate the discriminatory power of each VNTR locus and for the combination of the five loci for the Iranian and Australian isolates. This was calculated using the formula:

(1) $$1\;-\;\frac{1}{n\left(n\;-\;1\right)})\;\sum_{j\;=\;1}^{S}x_{j}\;\left(x_{j}\;-\;1\right)$$

where *n* is the total number of isolates tested, *S* the number of different genotypes and *xj* is the number of isolates belonging to the *j*th genotype [19]. The index was calculated using the Discriminatory Power Calculator available at http://insilico.ehu.es/mini_tools/discriminatory_power/.

The probability of a genotype occurring more than once in the dataset was determined using the formula

(2) $$\sum_{x\;=\;n}^{G}\;\frac{G!}{x!\left(G-x\right)!}{\left(P\right)}^{x}{\left(1\;-\;P\right)}^{G-x}$$

where *G* is the number of genotyped isolates in the population, *P* is the probability of the observation of the genotype in question (which is the product of the frequency of each allele found at a locus) and *n* is the number of isolates with the same genotype as that in question. In the case of pairs of identical genotypes, *n* = 1 and the formula reduces to

$$\mathrm{Pse}=\;1\;–\;{\left(1\;–\;P\right)}^{G}$$

[20].

### Analysis of genetic structure

Initial analysis of the MLV data used MICROSAT version 1.4 to calculate pairwise population distances using the proportional shared allele distance measure (DPs) [21]. Null alleles were scored as missing data. Pairwise distances were used to construct dendrograms using the Neighbour-Joining algorithm [22] available in the program PHYLIP 3.5c Bootstrap analyses were performed in MICROSAT with 1,000 replications. *A. parasiticus* reference strains were used as the outgroup.

To further analyze the structure of the Iranian population and to integrate this with other isolates a network-based approach was used using goeBURST with Minimum Spanning Tree implemented in PHYLOVIZ 1.0 [23] (available at http://goeburst.phyloviz.net/#Software). Isolates were assigned multilocus variable-number tandem-repeat (MLV) genotypes based on their VNTR data (Table 1) and these were integrated with taxonomic, geographic, clinical and toxin data to visualize and assess the relationships among strains.

## Results

### Iranian clinical isolates are diverse with some clonally related groups

VNTR loci AFPM-1, -4, -5, -6 and -7 were successfully amplified from the strains included in the initial analysis of Iranian and Australian isolates, yielding single bands in the expected size range (Table 1). Occasionally a VNTR locus could not be amplified from an isolate, and these were scored as null alleles and treated as missing data. Difficulties were encountered amplifying loci AFPM-2 and AFPM-3, which gave poor or no amplification or extra, spurious bands. Since this occurred with DNA from Australian isolates that had previously amplified with the primers specific to these loci [3] it was assumed that this was not due to genomic differences in this group of isolates, but that there were problems with primer degradation or sensitivity. As sufficient diversity was seen with the remaining five VNTR loci (Table 2), AFPM 2 and AFPM-3 were omitted from further analysis. The VNTR markers revealed a high degree of diversity among the Iranian isolates. Forty-eight unique multilocus variable-number tandem-repeat (MLV) genotypes were observed among the 58 isolates. Simpson’s Index of Diversity for the combined MLV was 0.9913 (Table 2).

**Figure 1:**
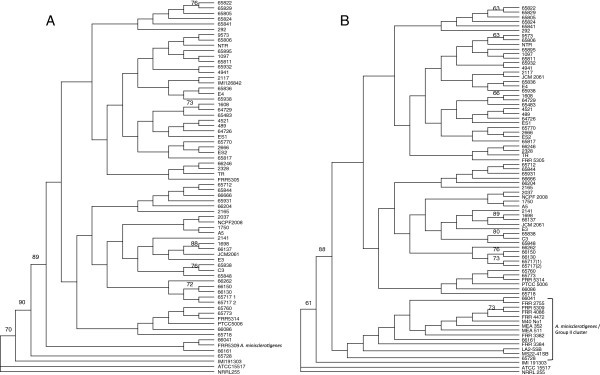
**Neighbor-joining phylograms of clinical and environmental *Aspergillus flavus *isolates based on MLV data. A)** Iranian and Australian isolates; **B)** Inclusion of additional Group II/*A. minisclerotigenes* isolates. Iranian clinical isolates 66401 and 66161 lie within the Group II/*A. minisclerotigenes* group, while isolate 65728 lies on the outside of this group.

Neighbour joining (NJ) and parsimony analyses conducted on the MLV dataset were used to construct preliminary dendrograms of strain relatedness. Dendrogram structure was largely congruent, and the NJ dendrogram is shown in Figure 1A. Considerable diversity is apparent among the Iranian isolates, and there is no obvious partitioning according to source (clinical or environmental) or type of infection. A number of clonally related groups containing isolates with identical MLV genotypes are apparent. Four of these groups include both clinical and environmental Iranian isolates, and one clonal group consists of an Iranian clinical isolate (65838) and an Australian clinical isolate (C3). Based on the frequency of the alleles that the clonally related isolates groups share (equation 2, above), all but two isolate pairs (Iranian isolates 9573 & 65806, and 66150 & 65130) were unlikely to appear to be clonally related by chance (p < 0.05; Table 3), and it therefore appears that these groups contain genetically identical isolates.

**Table 3:** Probability of isolates having identical MLV genotypes

**MLV group**	**Isolates**	***Pse***
1	65822^1^, 65829^1^, 65805^1^ & 65824^2^	0.022
3	9573^1^ & 65806^2^	0.24
7	1608^1^ & 64729^1^	0.03
20	1698^1^ & 66137^1^	0.031
24	65383^1^ & C3^3^	0.046
29	4521^1^, 489^1^, 64726^1^, & ES1^2^	0.0098
34	66150^1^ & 65130^2^	0.28

^1^Iranian clinical isolate.

^2^Iranian environmental isolate.

^3^Australian clinical isolate.

### Some Iranian clinical isolates group with *A. minisclerotigenes*

Two Iranian clinical isolates (66161 and 66041) grouped on the dendrogram with isolate FRR 5309, which has been characterized as *A. minisclerotigenes*[10], and one isolate (65728) lay outside the entire *A. flavus* group. To determine the relationship of these isolates with *A. minisclerotigenes,* ten isolates that were previously categorized as *A. flavus* Group II [3] and are likely to be *A. minisclerotigenes*[10] were added to the analysis (Table 1).

Figure 1B shows the dendrogram drawn using the MLV data for the Iranian collection with the addition of the *A. flavus* Group II isolates. From this it was clear, first that most isolates previously characterized as Group II lie in a monophyletic cluster that contains *A. minisclerotigenes* isolate FRR 5309; and second, that the Iranian clinical isolates 66161 and 66041 fit within this same cluster. Iranian isolate 65728 lay on the outside of the *A. minisclerotigenes* group. This isolate shares similar VNTR alleles with the Group II isolates but has a 123 bp allele for AFPM-1 while all other Group II isolates examined to date have a 119 bp allele at this locus (Table 1; Tran-Dinh 2002) [3].

### Microscopy and toxin analysis of *A. minisclerotigenes-*like isolates 66161, 66041 and 65728

Microscopic analysis of isolates 66161, 66041 and 65728 revealed these to have metulae, phialids and smooth spores indistinguishable from those of *A. flavus* (Additional file 1: Figure S1)*.* This is typical for *A. minisclerotigenes,* which is only morphologically distinguished from *A. flavus* by the production of small sclerotia [10].

*A. minisclerotigenes* isolates are known to produce both B and G aflatoxins, unlike *A. flavus* which can only make B aflatoxin. Bright blue-white fluorescence indicative of B and G aflatoxin was produced by isolate 66161 when grown on CCA, while 65728 and 66041 produced a moderate level of fluorescence. LC-MS was undertaken to confirm toxin analysis in these isolates (Table 4). 66161 was confirmed as a robust aflatoxin producer. Isolates 65728 and 66041 produced B and trace levels of G aflatoxins. All of the toxin profiles were dominated by aflatoxin B1.

**Table 4:** Aflatoxin produced following liquid culture in CCA (ng/mL)

**Isolate**	**B1**	**B2**	**G1**	**G2**
66161	12 498.2	6 598.2	19.6	27.8
66041	1 614.7	161.1	0.4	0.9
65728	7 002.3	562.9	0.4	0.4

### Integration of MLV genotypes with isolate data using goeBURST network analysis

goeBURST networks, drawn using the Minimum Spanning Tree expansion implemented in PHYLOVIZ [23] were used to visually integrate the MLV genotypes (Table 1) with taxonomy, clinical/environmental source and toxigenicity. Networks were initially drawn at single-, double-, and triple-locus variant levels (Additional file 2: Figure S2). All MLV genotypes were connected at the triple-locus variant level (TLV; Additional file 2: Figure S2A). The double-locus variant (DLV; Additional file 2: Figure S2B) level resolved type strains of *A. parasiticus* and *A. sojae* into a separate cluster and also separated the *A. minisclerotigenes*-like isolates, including Iranian clinical isolates 66041 (MLV genotype 16) and 66161 (MLV genotype 33), into a distinct cluster. Iranian isolate 65728 (MLV genotype 28), which was outside the *A. minisclerotigenes* group on the dendrogram (Figure 1B), did not group with other *A. minisclerotigenes*-like isolates and separated from all other isolates at this level. The single-locus variant (SLV) level (Additional file 2: Figure S2C) resolved *A. parasiticus* and *A. sojae* and separated *A. tamarii* from the remaining *A. flavus* isolates.

**Figure 2:**
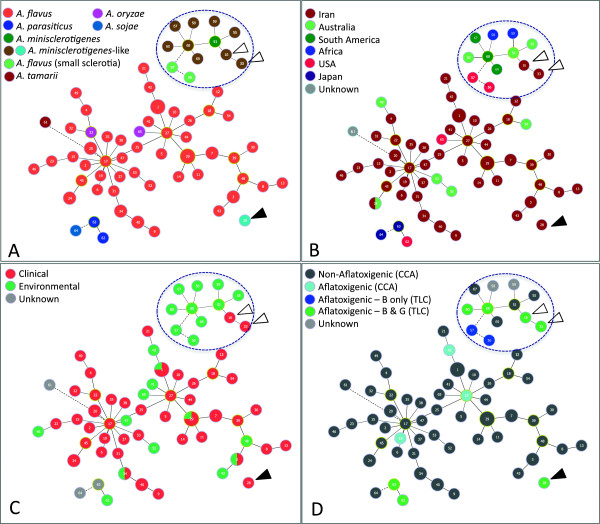
**goeBURST networks of clinical and environmental *Aspergillus flavus *isolates.** Networks were drawn using the Minimum Spanning Tree expansion implemented in PHYLOVIZ**.** Circles represent MLV genotypes and are proportional to the number of isolates with the same genotype; MLV genotypes are numbered as shown in Table 1. Solid lines show genotypes joined at the double locus variant (DLV) level; dashed lines show genotypes joined at the single locus variant (SLV) level. Networks are integrated with **A)** taxonomic; **B)** geographic; **C)** clinical; and **D)** toxin data. White arrows: Iranian *A. minisclerotigenes* isolates 66041 and 66161; black arrow: Iranian isolate 65728.

As the resolution of closely related species groups occurred at DLV level, with complete separation of species at the SLV level, Figure 2 shows the network drawn at the SLV level, with dotted lines connecting MLV genotypes that were joined at the DLV level. On this network, MLV genotypes are integrated with taxonomic (2a), geographic (2b), clinical/environmental source (2c) and toxigenicity (2d) data. Taxonomy of the isolates is clearly resolved, reinforcing the association of Iranian *A. minisclerotigenes*-like isolates 66041 and 66161 (white arrowheads) with the *A. minisclerotigenes* group; however on the network it is apparent that isolate 65728 (black arrowhead), which lay on the outside of the *A. minisclerotigenes* cluster on the dendrogram (Figure 1) is not related to *A. minisclerotigenes* and may be different to the other species included in the analysis.

There was no obvious clustering of isolates on the network in relation to geographic origin (Figure 2B), source (Figure 2C) or toxigenicity (Figure 2D). However, the two USA Group II/*A. minisclerotigenes*-like isolates (LA-5 SB; MLVA genotype 56 and MS22-41SB; MLVA genotype 57) that are known to produce B aflatoxin only separated from the rest of the *A. minisclerotigenes-*like group at the SLV level (Figure 2D, Additional file 2: Figure S2C).

### Phylogeny confirms two clinical isolates from Iran are *A. minisclerotigenes*

**Figure 3:**
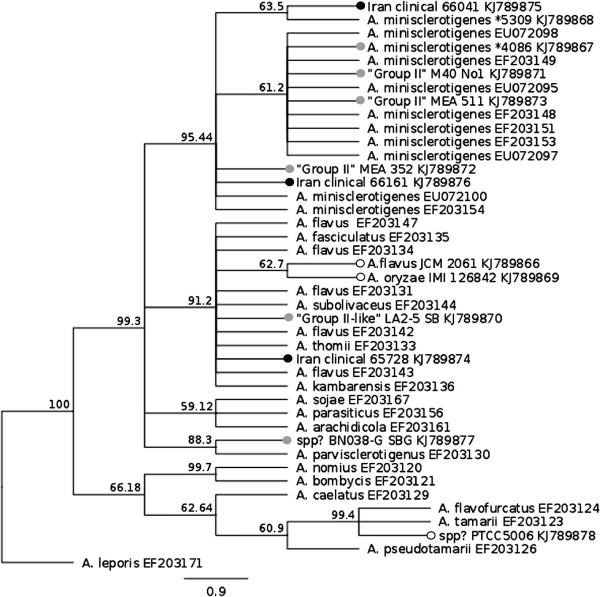
**Phylogenetic placement of Group II/*A. minisclerotigenes *-like isolates in *Aspergillus *section *flavi.*** A 444 bp fragment of the β-tubulin gene was amplified from Group II/*A. minisclerotigenes*-like isolates and aligned with published *Aspergillus* section *flavi* sequences from [10], using *A. leporis* as an outgroup. Maximum likelihood trees were drawn using the PAUP 4.0 plug-in for Geneious R6.1.6; bootstrap support was obtained using 1000 replicates. Isolates sequenced in this study are indicated by a dot at the terminus of their branch: white = strains from culture collections; grey = Group II isolates from our collection; black = clinical Iranian isolates. Most of the Group II isolates from our collection along with Iranian clinical isolates 66041 and 66161 form a distinct cluster with 95% bootstrap support with isolates previously characterized as *A. minisclerotigenes*[10], including isolate FRR5309. Iranian clinical isolate 65728 groups away from this cluster with *A. flavus* and closely related species. Group II-like isolates LA 2–5 SB and BN038 G SGB group within the *A. flavus* cluster and with *A. parvisclerotigenes,* respectively. Accession numbers for sequences are listed after the name of the relevant isolate. *denotes FRR.

β-tubulin and calmodulin gene sequences were used to confirm the phylogenetic relationship of Group II/*A. minisclerotigenes*-like isolates with species within *Aspergillus* section *Flavi* (Figure 3 and Additional file 3: Figure S3). On both trees the majority of the Group II/*A. minisclerotigenes*–like isolates, including Iranian clinical isolates 66041 and 66161, formed a distinct cluster with 71% (calmodulin) and 95% (β-tubulin) bootstrap support that contained characterized *A. minisclerotigenes* isolates [10], including FRR5309. Iranian clinical isolate 65728 grouped away from this cluster with other close relatives of *A. flavus*. Also notable on this tree, Iranian *A. flavus* type strain PTCC5006 from the Persian Type Culture Collection grouped with *A. tamarii* and *A. flavofurcatus*. This was consistent with the morphology of this isolate (Denghan, unpublished) and it appears that it has either been misidentified or has been inadvertently swapped with an *A. flavus* isolate. Group II-like isolate LA2-5 SB, which separated from other *A. minisclerotigenes*-like isolates on the goeBURST network at the SLV level (Figure 2; Additional file 2: Figure S2C) also did not cluster with *A. minisclerotigenes* isolates in the two phylogenies. Finally, isolate BN038-G SBG, which is characteristic of “S_BG_” isolates from West Africa that have yet to be classified [24], grouped with *A. parvisclerotigenes*[25] on both phylogenies with high bootstrap support.

### Both mating types are present in *A. minisclerotigenes*

Recent studies have established that *A. fumigatus* and *A. flavus* are sexual species with two mating types, MAT-1 and MAT-2, and primers have been developed to amplify these loci from different *Aspergillus* species [16]. Amplification of the *A. minisclerotigenes* isolates in our collection found both mating types to be present, although the MAT-1 mating type dominated (Additional file 4: Figure S4; Table 1). The two Iranian *A. minisclerotigenes* isolates were of opposite mating type. Although not direct evidence for sexual recombination, this indicates that recombination may be possible in the Iranian *A. minisclerotigenes* population. Two of the *A. flavus* type culture isolates, NCPF 2008 (from the National Collection of Pathogenic Fungi, UK) and JCM 2061 (from the Japanese Collection of Microorganisms) had both MAT-1 and MAT-2 alleles, indicating diploidy or aneuploidy at this locus and the possibility of homothallism. Iranian isolate 65728 and the six remaining *A. minisclerotigenes* isolates had the MAT-1 allele.

### Drug susceptibility of *A. minisclerotigenes* is similar to that of *A. flavus*

Susceptibility testing using CLSI microbroth dilution methods was performed on all *A. minisclerotigenes* isolates in our collection and on five clinical and one environmental Iranian *A. flavus* isolates, with the results shown in Table 5 (for complete dataset see Additional file 5). Epidemiological cut-off values (ECVs) have been established for the response of *A. flavus* populations to antifungal drugs as AMB: 2; ITR: 1; VOR: 1 and CAS (MEC): 0.06 [26-28]. All of the isolates tested had MIC values equal to or less than the ECVs for each drug, indicating that they do not have unusual drug susceptibilities. There were no significant differences between the MIC values for *A. flavus* and *A. minisclerotigenes.*

**Table 5:** **Antifungal MIC values for *A. flavus *** **and *A. minisclerotigenes***

**Species**	**AMB (MIC; μg/mL)**		**ITR (MIC; μg/mL)**		**VRZ (MIC; μg/mL)**		**CAS (MEC; μg/mL)**	
	**Geomean**	**Range**	**Geomean**	**Range**	**Geomean**	**Range**	**Mode**	**Range**
*A. flavus* (n = 8)^1^	**0.26**	0.06-1	**0.13**	0.03-0.25	**0.49**	0.25-1	**0.015**	<0.015-0.03
*A. miniscleotingenes* and Group II-like (n = 6)^2^	**0.3**	0.125-0.5	**0.1**	0.03-0.25	**0.37**	0.25-0.5	**0.015**	<0.015-0.03

^1^Includes 6 Iranian and 2 clinical Australian isolates.

^2^Includes Iranian isolates 66041, 66161 and 65728.

## Discussion

*A. flavus* is a particularly important fungus in human health, both through the production of highly toxic and carcinogenic secondary metabolites and through infection of immunocompromised and immunocompetent people. There are more than 25 described species and varieties within the *Aspergillus* section *Flavi*[7,10,29], but while all of these can produce various toxic metabolites very few cause human infection. *A. flavus*, as the second most common cause of invasive aspergillosis behind *A. fumigatus,* and the cause of most superficial, cutaneous, ocular and sinus *Aspergillus* infections, is by far the most medically important species in this group [7]. *A. oryzae*, which is a non-toxigenic morphological variant of *A. flavus*, causes occasional disease, and there are single reports of clinical infection caused by *A. tamarii, Petromyces alliaceus, A. qizutongi* and *A. beijingensis*[7]. In this study we report for the first time human infection caused by *A. minisclerotigenes*, a recently described species within *Aspergillus* section *Flavi* and closely related to *A. flavus*[10]. With two clinical isolates among a collection of 46, *A. minisclerotigenes* may be a previously unrecognized cause of aspergillosis in Iran.

*A. minisclerotigenes* was first described as “*A. flavus* Group II” by Geiser et al. (1998) and formed a distinct clade within a collection of *A. flavus* isolates from Australian peanut growing soils [9]. Subsequent analyses found fungi in this group were capable of producing B and G aflatoxins as well as cycolpiazoic acid (CPA), a chemotype that distinguished them from *A. flavus*, which produces B aflatoxins and CPA, and *A. parasiticus*, which produces B and G aflatoxins but not CPA. These fungi were also characterized by the production of small sclerotia, leading to their eventual formal description as *A. minisclerotigenes*[10]. Like *A. flavus, A. minisclerotigenes* produces copious readily aerosolized conidia (Additional file 1: Figure S1), however, although it has been found sympatrically with *A. flavus* at relatively high levels in crops and soils from Australia, West Africa, Argentina, Algeria and Portugal [29,30], it is rare or absent from similar soil types sampled in the United States, India and Asia, and prior to this study it had never been reported from East Africa or the Middle East [9,31-33]. This suggests that dispersal in these two species is different or that *A. minisclerotigenes* is regulated by local factors that act to suppress or enhance its growth.

The current study identified a third clinical isolate (65728) that was distinct from both *A. flavus* and *A. minisclerotigenes* on the goeBURST networks (MLV type 28; Figure 2) but could not be distinguished from *A. flavus* and related species by β-tubulin and calmodulin gene sequencing (Figure 3 and Additional file 3: Figure S3). This isolate produced B and trace levels of G aflatoxin and may be another, as yet undescribed species within *Aspergillus* section *Flavi*. Given its ability to cause disease it would be interesting to know whether related isolates can be obtained from the environment in Iran.

As clinical disease can be caused by *A. minisclerotigenes* it was important to determine how this species responds to antifungal drugs used to treat *Aspergillus* infections. We therefore tested all of the *A. minisclerotigenes* isolates in our collection, along with six clinical and one environmental Iranian *A. flavus* isolates and the MAT-1/MAT-2 clinical *A. flavus* isolate NCPF2008, to four commonly used antifungals. Antifungal breakpoints have yet to be established for *A. flavus*, but large collections of wild-type isolates (defined as having no acquired or mutational mechanisms of resistance) have been used to determine epidemiological cut-off values (ECVs) for AMB [26], CAS [28] and the triazoles [27], which can be used to identify isolates with unusual drug susceptibility. All of the *A. minisclerotigenes* isolates were below the ECVs for each of the tested antifungals. Likewise the clinical and environmental Iranian *A. flavus* isolates had MICs below the established ECVs and appear to be similar in susceptibility to global *A. flavus* isolates. It therefore appears that the antifungal regimes that are currently used for *A. flavus* are applicable to *A. minisclerotigenes* and to *A. flavus* infections in Iran, and it is not necessary to differentiate between these two species in order to treat clinical disease.

In the Middle East and India, *A. flavus* is not only the leading cause of superficial aspergillosis but it can also surpass *A. fumigatus* as the cause of invasive disease [34]. Exactly why Iran and other countries in the Middle East have a much higher incidence of disease due to *A. flavus* than is seen in other regions is not fully understood. Generally it is assumed that the hot and dry local climate favours growth of desiccation- and thermo-tolerant species including *A. flavus*, and their higher environmental presence translates into a higher likelihood of infection [35]. However, parts of Australia have similar climatic conditions, and a recent survey found Australian peanut growing soils to be exceptionally high in *A. flavus* propagules, with a mean of >5,000 (SD > 20,000) CFU/g [33], yet *A. flavus* is a rare cause of infection in Australia. In the current study we hypothesized that fungal factors might underlie these differences and therefore that Iranian clinical isolates might be genetically distinct from their environmental counterparts, and from clinical and environmental isolates from Australia. goeBURST analysis of VNTR loci was unable to detect any differentiation among these groups, however, and in fact some genetically identical isolates were found across these groups (Figure 2; Table 3). This is in agreement with other studies that have found *A. flavus* to have a globally panmictic population structure [3,4,36], although at very local scales, such as single hospitals or farms, single clades can dominate [5,36]. It is a limitation of our study that we did not include more Australian or global isolates, as we had initially assumed we would be able to combine this analysis with a global analysis already performed by Tran-Dinh (2002) [3]. Unfortunately, differences were noted in how bands were sized following a change in equipment at the SUPAMAC facility and it was not possible to combine datasets. This problem has been noted by other researchers and is an issue for establishing on-line accessible databases for sharing MLV data [37]. Nonetheless, from our data it seems reasonable to conclude that clinical *A. flavus* isolates from Iran are not notably distinct from environmental or Australian strains.

There was likewise no discernible differentiation between toxigenic and non-toxigenic isolates, although it was noted that in this collection there is a paucity of toxigenic isolates, with only five for the 54 (9.25%) clinical and environmental isolates testing positive for aflatoxin. This is very similar to a recent study of *A. flavus* from Iranian hospital environments, in which ~10% were aflatoxigenic [1], and contrasts with findings from other studies, where 20 - >90% of isolates from Australia, Asia and the Americas have been found to be aflatoxigenic [3,31,33,38]. It also differs to surveys of *A. flavus* from corn and peanut-growing soils in Iran, in which 27.5% and 85% of isolates, respectively, produced B aflatoxins [6,39]. Different laboratory growth and assay conditions can influence the extent of toxin formation, however studies from our laboratory have used the same toxin assays but found other populations to contain more toxigenic isolates [3,31], which suggests the level of aflatoxigenic isolates in this clinical collection may be genuinely lower than in most regions and in Iranian soils. Interestingly, TLC analysis of toxin production by isolates in this collection by Denghan et al. [8] found isolates 66041 and 66161 (designated isolates 20 and 40, respectively, in their paper and here identified as *A. minisclerotigenes*), produced the highest levels of B aflatoxin. Further analysis of Iranian crops and soil are needed to determine if *A. minisclerotigenes* is a significant source of aflatoxin contamination in Iranian foodstuffs.

The study of *Aspergillus* taxonomy and the role of aflatoxigenic species in clinical disease have important implications for biocontrol strategies that aim to control aflatoxin contamination of crops. These strategies rely on competitive exclusion using non-aflatoxigenic strains, which are seeded into fields to prevent colonization and growth of native toxigenic strains [40-42]. While these have shown promise in reducing aflatoxin levels in the short term, the current study and others that have examined the diversity of clinical *A. flavus* isolates indicate that potentially any *A. flavus* strain could cause infection in people and animals, and this could include non-aflatoxigenic biocontrol strains. The use of a closely related non-pathogenic species might reduce this risk, but it is possible that like *A. minisclerotigenes*, other close relatives of *A. flavus* can cause infection. There is currently very little known about what determines mammalian virulence in *A. flavus*[35]. Clearly these studies are essential before the safety of biocontrol strains can be assured.

## Conclusion

We report that clinical *A. flavus* isolates from Iran are not significantly different from environmental or Australian isolates, and that local geographic, climatic or host factors are more likely than fungal factors to govern the relatively high incidence of disease in this region. We report for the first time that *A. minisclerotigenes* can cause human infection and that this species may also be an important cause of aflatoxin contamination. Treatment of *A. minisclerotigenes* infection can be undertaken using the same antifungal regime recommended for *A. flavus*. The possibility of clinical infection must be taken into account when choosing *A. flavus* strains and related species for control of aflatoxin in crops.

## Abbreviations

VNTR: Variable number tandem repeat; MLV: Multilocus variable-number tandem repeat; MLVA: MultiLocus variable-number tandem repeat analysis; SLV: Single locus variant; DLV: Double locus variant; TLV: Triple locus variant; CLSI: Central Laboratory Standards Institute; AMB: Amphotericin B; ITZ: Itraconazole; VRZ: Voriconazole; CAS: Caspofungin; ECV: Epidemiological cut-off values.

## Competing interests

The authors declare that they have no competing interests.

## Authors’ contributions

PD collected and identified the isolates from Iran, carried out the toxin and VNTR analyses and helped to draft the manuscript; TB assisted with the VNTR analysis and undertook the MICROSAT analysis; LC performed the phylogenetic and mating type analyses; NT-D performed microscopy and toxin analysis on *A. minisclerotigenes* isolates; Y-WL performed the antifungal susceptibility testing; FZ assisted with the collection and morphological identification of isolates; DC conceived of, designed and coordinated the study, undertook the goeBURST analysis and wrote the manuscript. All authors read and approved the final manuscript.

## Pre-publication history

The pre-publication history for this paper can be accessed here:

http://www.biomedcentral.com/1471-2334/14/358/prepub

## Supplementary Material

Additional file 1: Figure S1 Light microscopy of conidial heads and conidia from Iranian *A. minisclerotigenes*/Group II-like isolates. A) Strain 66041; B) Strain 66166; C) Strain 65728. Click here for file

Additional file 2: Figure S2 goeBURST networks of clinical and environmental *A. flavus* isolates. Networks are drawn at the A) triple-; B) double-; and C) and single- locus variant levels. MLV genotypes are as shown in Table 1. Separation of different *Aspergillus* species occurs at the double- and single-locus variant levels. Click here for file

Additional file 3: Figure S3 Phylogenetic placement of *A. minisclerotigenes*-like isolates using partial sequence of the calmodulin gene. Maximum likelihood trees were drawn using the PAUP 4.0 plug-in for Geneious R6.1.6; bootstrap support was obtained using 1000 replicates. Isolates sequenced in this study are indicated by a dot at the terminus of their branch: white = strains from culture collections; grey = Group II isolates from our collection; black = clinical Iranian isolates. Most Group II isolates and Iranian clinical isolates 66041 and 66161 form a cluster with isolates previously characterized as *A. minisclerotigenes*. Iranian clinical isolate 65728 groups with *A. flavus* and other closely related species. Accession numbers for sequences are listed after the name of the relevant isolate. *denotes FRR. Click here for file

Additional file 4: Figure S4 PCR amplification of mating type loci from *A. minisclerotigenes* and selected type strains. Top panel: Amplification with MAT-1 primers; bottom panel: Amplification with MAT-2 primers. **Lanes:** 1. NCPF2008; 2. JCM2061; 3. PTCC 5006; 4. NRRL 255; 5. IMI 126842; 6. FRR5309; 7. 66041; 8. 65728; 9. 66161; 10.FRR 4472; 11. FRR4086; 12. LA2-5 SB; 13. FRR 3384; 14. M40 N°1; 15. MEA511; 16; MEA 342; 17. –ve control. Click here for file

Additional file 5: Table S1 MIC data for *Aspergillus minisclerotigenes* and selected *A. flavus* isolates. Click here for file
