# Supplementary figures and images for: Multilocus variable-number tandem-repeat analysis of clinical isolates of Aspergillus flavus from Iran reveals the first cases of Aspergillus minisclerotigenes associated with human infection
Source: BMC Infect Dis. 2014 Jul 1;14:358. doi: 10.1186/1471-2334-14-358 (PMC4099206; doi:10.1186/1471-2334-14-358)

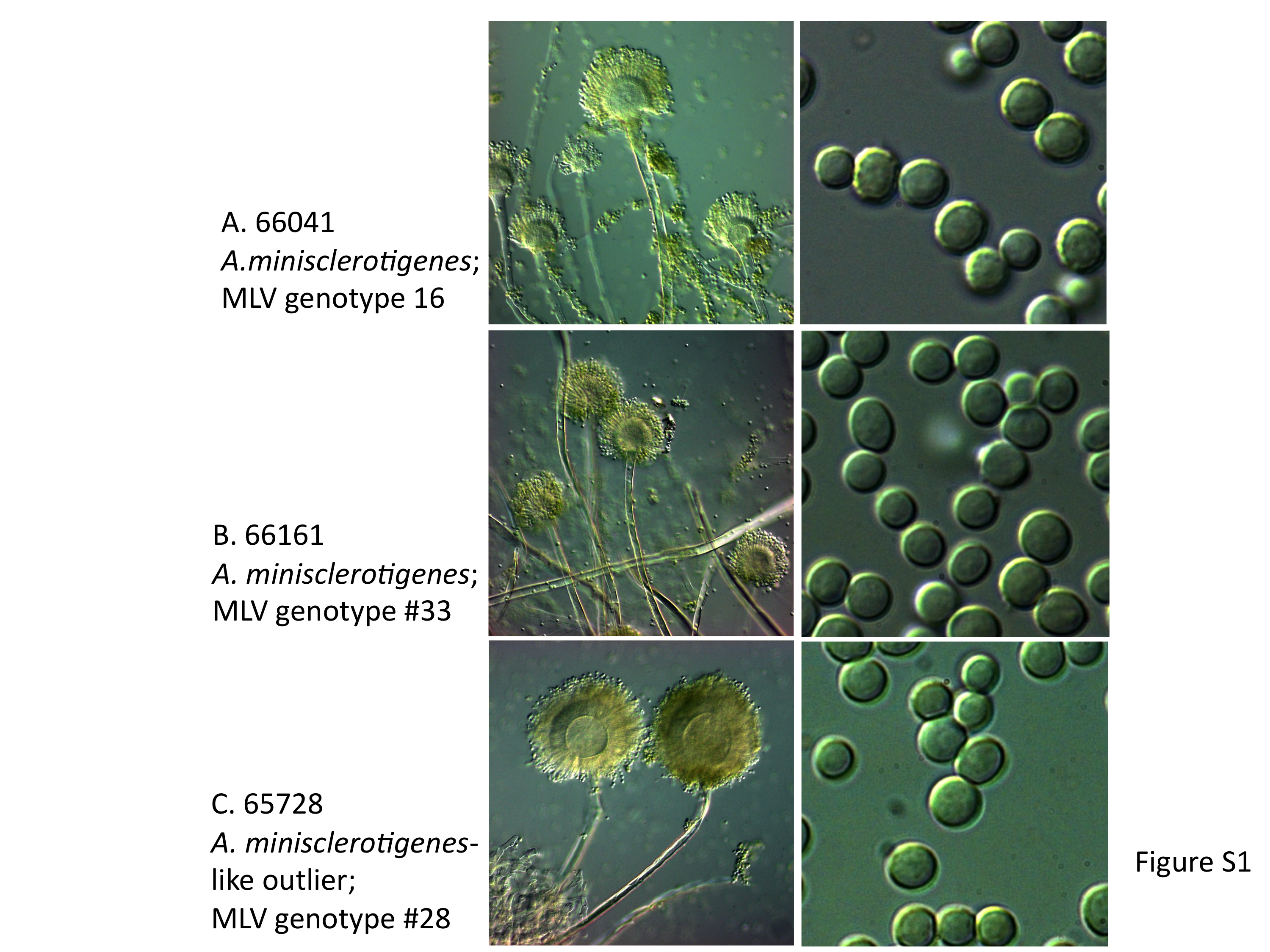

Supplement: Additional file 1: Figure S1 — Light microscopy of conidial heads and conidia from Iranian A. minisclerotigenes/Group II-like isolates. A) Strain 66041; B) Strain 66166; C) Strain 65728. [file 1471-2334-14-358-S1.png]

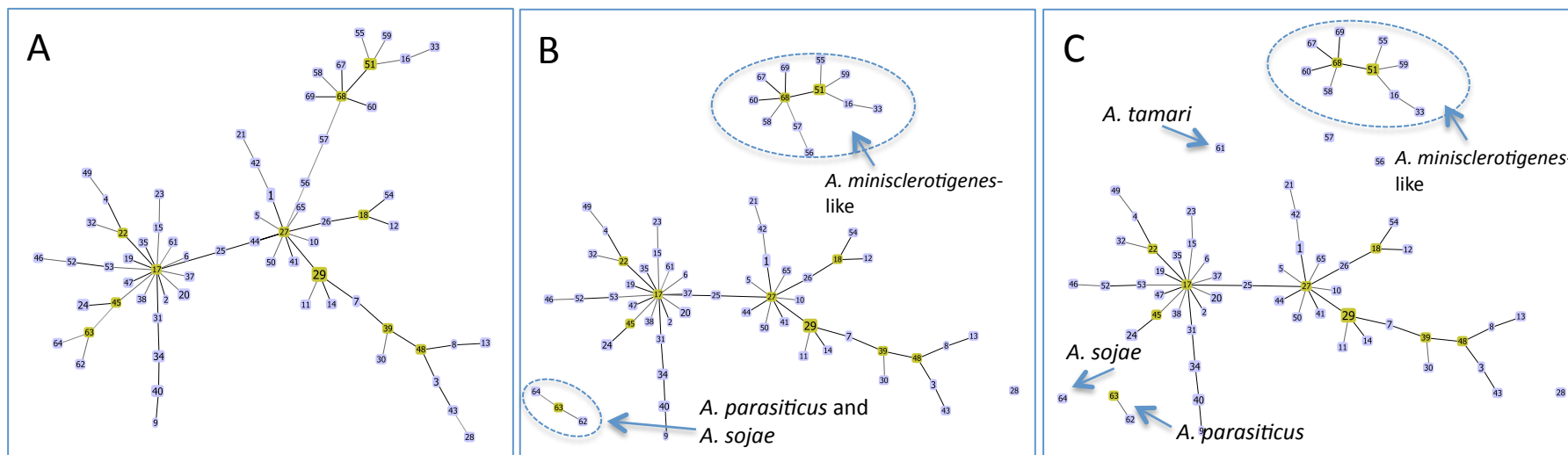

Figure S2

Supplement: Additional file 2: Figure S2 — goeBURST networks of clinical and environmental A. flavus isolates. Networks are drawn at the A) triple-; B) double-; and C) and single- locus variant levels. MLV genotypes are as shown in Table 1. Separation of different Aspergillus species occurs at the double- and single-locus variant levels. [file 1471-2334-14-358-S2.pdf]

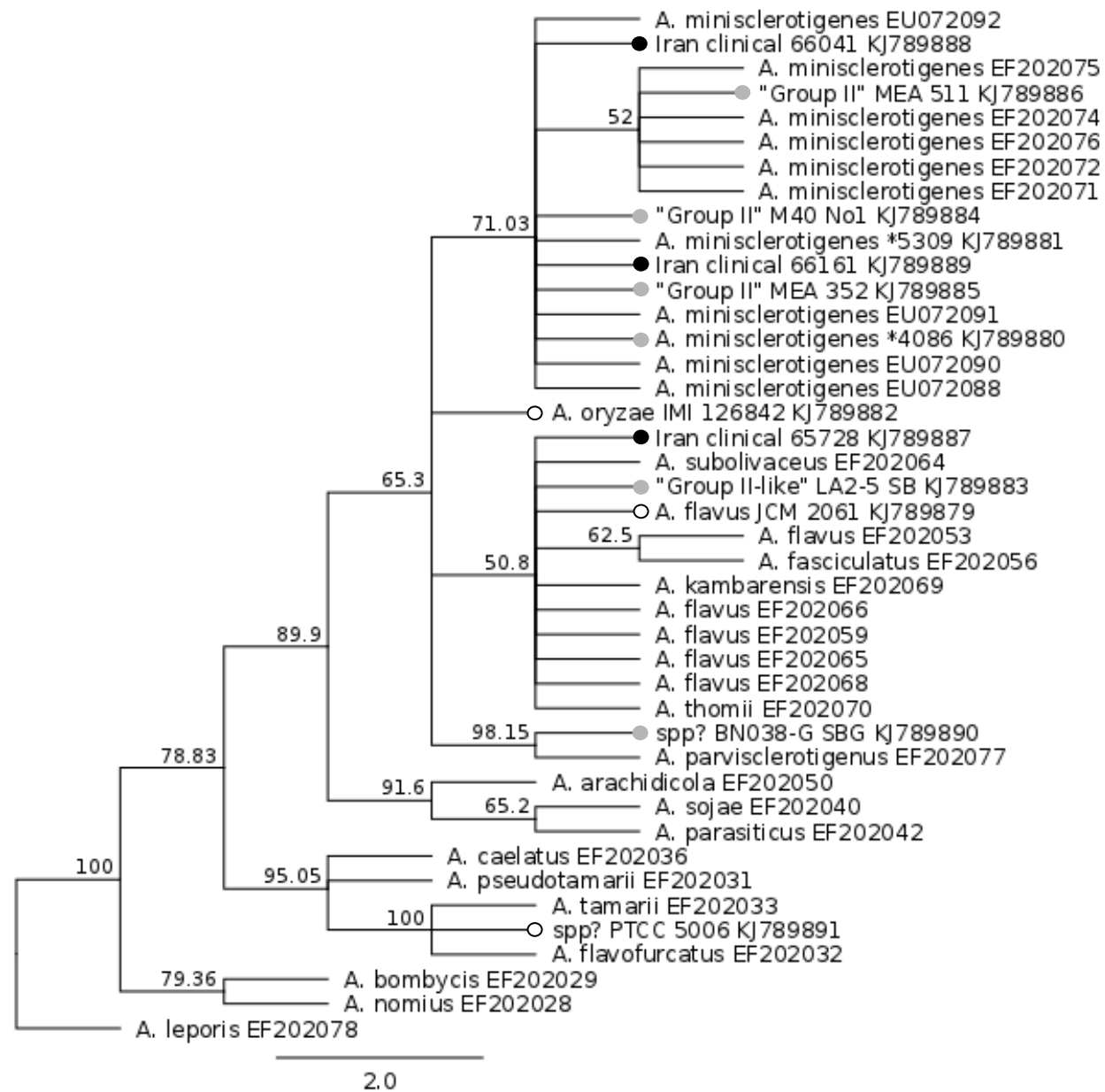

Figure S4

Supplement: Additional file 3: Figure S3 — Phylogenetic placement of A. minisclerotigenes-like isolates using partial sequence of the calmodulin gene. Maximum likelihood trees were drawn using the PAUP 4.0 plug-in for Geneious R6.1.6; bootstrap support was obtained using 1000 replicates. Isolates sequenced in this study are indicated by a dot at the terminus of their branch: white = strains from culture collections; grey = Group II isolates from our collection; black = clinical Iranian isolates. Most Group II isolates and Iranian clinical isolates 66041 and 66161 form a cluster with isolates previously characterized as A. minisclerotigenes. Iranian clinical isolate 65728 groups with A. flavus and other closely related species. Accession numbers for sequences are listed after the name of the relevant isolate. *denotes FRR. [file 1471-2334-14-358-S3.pdf]

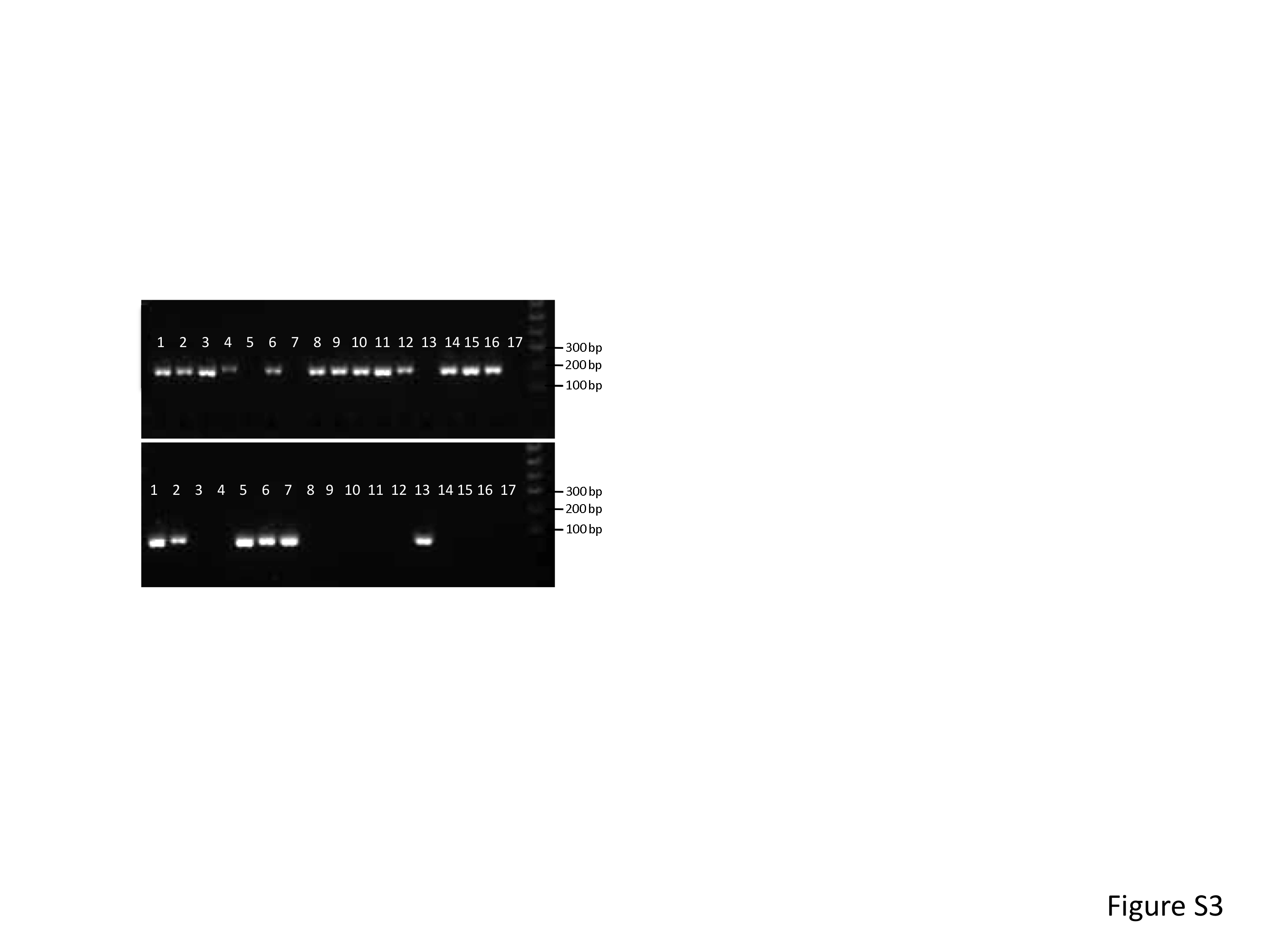

Supplement: Additional file 4: Figure S4 — PCR amplification of mating type loci from A. minisclerotigenes and selected type strains. Top panel: Amplification with MAT-1 primers; bottom panel: Amplification with MAT-2 primers. Lanes: 1. NCPF2008; 2. JCM2061; 3. PTCC 5006; 4. NRRL 255; 5. IMI 126842; 6. FRR5309; 7. 66041; 8. 65728; 9. 66161; 10.FRR 4472; 11. FRR4086; 12. LA2-5 SB; 13. FRR 3384; 14. M40 N°1; 15. MEA511; 16; MEA 342; 17. –ve control. [file 1471-2334-14-358-S4.png]
